# Development of Dental Poly(methyl methacrylate)-Based Resin for Stereolithography Additive Manufacturing

**DOI:** 10.3390/polym13244435

**Published:** 2021-12-17

**Authors:** Kentaro Hata, Hiroshi Ikeda, Yuki Nagamatsu, Chihiro Masaki, Ryuji Hosokawa, Hiroshi Shimizu

**Affiliations:** 1 Division of Oral Reconstruction and Rehabilitation, Department of Oral Functions, Kyushu Dental University, Fukuoka 803-8580, Japan; r17hata@fa.kyu-dent.ac.jp (K.H.); masaki@kyu-dent.ac.jp (C.M.); hosokawa@kyu-dent.ac.jp (R.H.); 2 Division of Biomaterials, Department of Oral Functions, Kyushu Dental University, Fukuoka 803-8580, Japan; yuki-naga@kyu-dent.ac.jp (Y.N.); r14shimizu@fa.kyu-dent.ac.jp (H.S.)

**Keywords:** methyl methacrylate (MMA), additive manufacturing, vat polymerization, mechanical property, physicochemical property, cytotoxicity, dental materials

## Abstract

Poly(methyl methacrylate) (PMMA) is widely used in dental applications. However, PMMA specialized for stereolithography (SLA) additive manufacturing (3D-printing) has not been developed yet. This study aims to develop a novel PMMA-based resin for SLA 3D-printing by mixing methyl methacrylate (MMA), ethylene glycol dimethacrylate (EGDMA), and PMMA powder in various mixing ratios. The printability and the viscosity of the PMMA-based resins were examined to determine their suitability for 3D-printing. The mechanical properties (flexural strength and Vickers hardness), shear bond strength, degree of conversion, physicochemical properties (water sorption and solubility), and cytotoxicity for L929 cells of the resulting resins were compared with those of three commercial resins: one self-cured resin and two 3D-print resins. EGDMA and PMMA were found to be essential components for SLA 3D-printing. The viscosity increased with PMMA content, while the mechanical properties improved as EGDMA content increased. The shear bond strength tended to decrease as EGDMA increased. Based on these characteristics, the optimal composition was determined to be 30% PMMA, 56% EGDMA, 14% MMA with flexural strength (84.6 ± 7.1 MPa), Vickers hardness (21.6 ± 1.9), and shear bond strength (10.5 ± 1.8 MPa) which were comparable to or higher than those of commercial resins. The resin’s degree of conversion (71.5 ± 0.7%), water sorption (19.7 ± 0.6 μg/mm^3^), solubility (below detection limit), and cell viability (80.7 ± 6.2% at day 10) were all acceptable for use in an oral environment. The printable PMMA-based resin is a potential candidate material for dental applications.

## 1. Introduction

The additive manufacturing (3D-printing) process is widely used to fabricate dental materials made of metals, ceramics, and resins [1]. Resin-based materials are becoming increasingly popular in the fabrication of various dental applications, including custom impression trays [2,3], denture base and teeth [4,5,6], provisional and temporary crowns and bridges [7], dental casts [8,9], wax patterns [10], and surgical implant guides [11]. The 3D-printing of resin-based materials can be carried out by various processes, including material extrusion, material jetting, powder bed fusion, and vat polymerization [12,13,14,15,16]. In material extrusion, thermoplastic resin is heated above its melting temperature, extruded from a nozzle head, and continuously deposited on a platform [13]. In the material jetting, a layer-by-layer structure is formed by material deposition directly onto the building platform via the print head, followed by curing via light exposure [13]. Powder bed fusion, which includes selective laser melting, can fabricate parts by laser sintering of polymer particles [13]. Vat polymerization, which includes stereolithography (SLA) and digital light processing (DLP), involves the layer-by-layer formation of an object using a photocurable resin and light exposure [17]. SLA 3D-printing allows rapidly fabrication of complex shapes with high accuracy and trueness. While SLA 3D-printing is mainly used to fabricate resin-based materials, it can also fabricate ceramic and metal objects, although additional de-binding and sintering steps after printing are mandatory for such objects [18].

Poly(methyl methacrylate) (PMMA) is one of the most commonly used resins for dental materials owing to its low cost, ease of processing, superior physicochemical properties, biocompatibility, and aesthetic features [19]. PMMA is frequently used for denture bases, denture teeth, temporary crowns, provisional restorations, orthodontic retainers, custom impression trays, and materials for prostheses repair [20,21,22]. PMMA has conventionally been shaped by dental technicians. However, computer-aided design/computer-aided manufacturing (CAD/CAM) has become an increasingly popular alternative to conventional processes in the design and fabrication of PMMA dentures [23]. Most commercially available systems use subtractive manufacturing to fabricate PMMA-based prostheses [24]. Furthermore, PMMA-based materials have been developed for 3D-printing processes. PMMA can be printed by the material extrusion 3D-printing owing to its high thermoplasticity. This process is widely used for a variety of applications, including dental materials [25,26]. PMMA-based materials for the SLA 3D-printing have been studied in the field of biomaterials, including bone tissue engineering [27]. However, for dental applications, the SLA 3D-printing of PMMA-based materials has not yet been established, although different acrylic resins are used in commercial products [28,29].

This study aims to develop a novel PMMA-based resin for SLA 3D-printing. To this end, we prepared a PMMA-based resin using cross-linkers, pre-polymerized PMMA powders, and photo-initiator. An SLA 3D-print resin typically contains multifunctional monomers in the form of photocurable resins with a photo-initiator [30]. The photo-initiator produces reactive radical species upon light irradiation, thereby initiating chain-growth polymerization, which forms a three-dimensional polymer network. Methyl methacrylate (MMA) is a monofunctional monomer that does not form a three-dimensional polymer network, making it difficult to curing by the SLA printing process without the addition of any multifunctional monomers. This composition system is similar to a traditional powder-liquid mixture system that is adapted to heat-cured and cold-cured PMMA. In this system, the pre-polymerized PMMA powders enhanced the curing speed. In this work, we prepared a PMMA-based resin with an optimal composition for SLA 3D-printing from appropriate amounts of MMA, pre-polymerized PMMA powder, and the multifunctional monomer ethylene glycol dimethacrylate (EGDMA). The mechanical, bonding, and physicochemical properties of the prepared resins were compared to commercially available 3D-print resins and conventional self-cured PMMA. The results are discussed in relation to the potential applications of the present materials.

## 2. Materials and Methods

### 2.1. Materials

The reagents used in the preparation of printable PMMA-based resins are listed in Table 1. Three commercial resins were used as comparison samples (Table 2): one conventional self-cured PMMA resin (UF) and two 3D-print resins used for denture base (DB) and teeth (DT).

### 2.2. Preparation of the PMMA-Based Resins for SLA 3D-Printing

Forty-three PMMA-based resins were prepared using the reagents listed in Table 3. PMMA powder was added to a liquid mixture of MMA and EGDMA monomers, and mixed using a magnetic stirrer (RCX-1000H, TOKYO RIKAKIKAI CO., LTD, Tokyo, Japan) at 300 rpm and 80 °C for 30 min. The resulting mixture was cooled to 25 °C before the photo-initiator, phenylbis(2,4,6-trimethylbenzoyl) phosphine oxide) (BAPO), was added using a planetary centrifugal mixer (ARE-310, THINKY Corp., Tokyo, Japan) at 2000 rpm for 2 min to yield the liquid PMMA-based resin used for SLA 3D-printing.

### 2.3. Viscosity Measurement for the PMMA-Based Resins

The viscosities of the prepared PMMA-based resins and commercial 3D-print resins (DB and DT) were measured using a B-type viscometer (ROTAVISC me-vi Complete, IKA^®^-Werke GmbH & Co. KG, Staufen, Germany) at 10–40 °C.

### 2.4. Sample Shape Design for 3D Printing

A 3D-printing model was designed using 3D-CAD software (Fusion 360, Autodesk Inc, San Rafael, CA, USA). A rectangular shape (4.5 mm × 14.5 mm × 2 mm) was used to evaluate the printability, three-point bending, Vickers hardness, water sorption and solubility, degree of conversion, and cell viability. A disk shape (diameter = 10 mm; thickness = 1.5 mm) was used in the shear bond strength (SBS) test. The models were exported in the standard triangulated language (STL) format. The STL data were imported into a software program (CHiTuBoX, CHITUBOX, Shenzhen, China) and converted to the slice data format for printing.

### 2.5. SLA 3D-Printing of the PMMA-Based Resin

Each group comprised 36 rectangular PMMA-based resin samples with a thickness of 0.01 mm fabricated by one-time printing using an SLA 3D-printer (ELEGOO MARS, λ = 400–410 nm 24 W LED laser, ELEGOO INC., Shenzhen, China) with an exposure time of 15 s. The printed samples were washed with MMA to remove any remaining uncured resin from the sample surface, and then post-cured with UV radiation from the top and bottom sides for 5 min using a UV-irradiator (LCR5N, J. MORITA Corp., Suita, Japan).

### 2.6. Sample Fabrication for Each Evaluation

The commercial 3D-print resins DB and DT were printed, washed, and post-cured in the same way as the PMMA-based resins. Conventional PMMA (UF) was formed into a silicone mold using a brush-dip technique according to the manufacturer’s instructions. Each sample (the PMMA-based resins, UF, DB, and UF) was polished using emery papers up to # 2000. The final dimensions of the rectangular samples were adjusted to 4 ± 0.2 mm × 14 ± 0.2 mm × 1.2 ± 0.2 mm. The disk samples measured 10 ± 0.1 mm in diameter and 1 ± 0.1 mm in thickness.

### 2.7. Three-Point Bending Test

Three-point bending tests were performed using rectangular samples (4 mm × 14 mm × 1.2 mm) using a universal testing machine (AGS-H, Shimadzu Corp., Kyoto, Japan) with a crosshead speed of 1 mm/s and a support span of 12 mm. The flexural strength (σ) was calculated using Equation (1):

σ = 3FL/2bh^2^ (1)

where F is the maximum load by the fracture, L is the support span, and b and h are width and thickness of the sample, respectively. Ten measurements were taken in each group (n = 10).

### 2.8. Vickers Hardness Test

The Vickers hardness test was performed using a micro-Vickers hardness tester (HMV-G21ST, Shimadzu Corp., Kyoto, Japan) with a load of 200 g and a dwell time of 15 s. Ten measurements were taken in each group (n = 10).

### 2.9. Shear Bond Strength Test

The SBS between eash sample and a commercial PMMA used in a self-cured resin (UNIFAST II A3, GC Corp., Tokyo, Japan) was measured to elucidate the bonding strength of the PMMA-based resins. SBS tests were performed as described in a previous study [31]. A Teflon tube (5 mm inner diameter, 3 mm height) was taped onto the sample surface to create a constant bonding area. The self-cured resin was loaded onto the sample surface using the brush-dip technique and kept at 25 °C for 1 h to enable polymerization. The Teflon tube was subsequently removed from the sample surface. Thermocycling was conducted using a thermocycle testing machine (K178, Tokyo Giken Inc., Tokyo, Japan) by alternately immersing the sample in water baths at 5 °C and 55 °C for 10,000 cycles with a dwell time of 60 s in each bath. The resulting sample was fixed to a jig and the SBS between the sample and the self-cured resin was measured using a universal testing machine with a crosshead speed of 1 mm/min. After the test, the de-bonded surface was examined using optical microscopy. Its failure mode was classified as an adhesive failure at the interface, cohesive failure within the sample, and mixed adhesive and cohesive failures.

### 2.10. Degree of Conversion

The degree of conversion of C=C bonds in the samples subjected to the Vickers hardness test was determined using Fourier transform infrared (FT-IR) spectroscopy (IRSpirit, Shimadzu Corp., Kyoto, Japan) with attenuated total reflectance. In the FT-IR spectra, two characteristic bands at 1637 cm^−1^ (C=C stretch) and 1720 cm^−1^ (C=O stretch) before and after polymerization of the sample were used to calculate the degree of conversion using Equation (2) [32]:

(2) $$\mathrm{Degree}\;\mathrm{of}\;\mathrm{conversion}\;\left(\%\right)=\left(1-\frac{{\left[A\left(C=C\right)/A\left(C=O\right)\right]}_{\mathrm{polymer}}}{{\left[A\left(C=C\right)/A\left(C=O\right)\right]}_{\mathrm{monomer}}}\right)\times 100$$

where A(C=C) and A(C=O) are their band intensities.

### 2.11. Physicochemical Properties

The physicochemical properties, water sorption, and water solubility of rectangular samples were measured using the procedures described in the ISO 10477:2018 standard. Prior to the measurement, all samples were maintained at 37 °C for seven days in an oven under dry conditions. Each sample was weighed using an electronic balance (m1) before being immersed in distilled water at 37 °C for seven days prior to being weighed again (m2). Subsequently, the sample was dried in an oven for seven days and weighed for a third time (m3). Equations (3) and (4) were used to calculate the water sorption (ρ_ws_) and solubility (ρ_sl_), respectively:

(3) $$\rho_{\mathrm{ws}}=\frac{\left(m2-m3\right)}{V}$$

(4) $$\rho_{s1}=\frac{\left(m1-m3\right)}{V}$$

where *V* is the volume of the rectangular sample. Ten measurements were taken in each group.

### 2.12. Cell Viability

For the cell viability test, each sample (the PMMA-based resin, self-cured resin, and commercial 3D-print resins) was cleaned in an ultrasonic bath with distilled water and ethanol for 10 min before being rinsed with 80% ethanol. The cleaned samples were dried and sterilized by exposure to UV light for 2 h on both the top and bottom sides.

The cell viability test was performed using fibroblast cell culture (L-929 mouse fibroblast, ECACC 85011425, ECACC, Wiltshire, UK). L-929 cells have previously been used for cytotoxicity testing of dental materials [33,34]. The cells were cultured according to the manufacturer’s recommendations and standard cell culture and maintenance protocols. The cells were cultured in cell culture growth medium containing Dulbecco’s modified Eagle’s medium (DMEM, D-MEM (High Glucose), Fujifilm, Tokyo, Japan) with 10% fetal bovine serum (Corning, NY, USA) and 1% penicillin/streptomycin (Penicillin-Streptomycin Solution (×50), Fujifilm, Tokyo, Japan). All procedures were performed aseptically, and all incubations were conducted in an incubator at 37 °C in a humid atmosphere comprised 5% CO_2_ and 95% air. The cells were expanded and passaged at regular periods based on their growth characteristics and manufacturer’s protocol. Cells were trypsinized on achieving confluence (0.25% *w/v* trypsin-EDTA, Fujifilm, Tokyo, Japan). After counting the cells with a hemocytometer, 5 × 10^4^ cells were seeded into each well of sterile 24-well tissue culture plates in complete growth medium (1 mL, 5 × 10^4^ cells/mL). After 24 h, at which time the cells were attached, each sterilized sample was placed in the center of a well, which signified the start of the test. The cells were cultured for 1, 3, 5, and 10 d to examine cell viability using a colorimetric assay (CCK-8 solution; Cell Counting Kit-8, Dojindo, Kumamoto, Japan). After culturing for a specified period, CCK-8 solution (40 μL) was added to each well and colorization was performed in incubator at 37 °C for 1 h. The supernatant (100 μL) was then removed from each well and transferred to a 96-well plate for analysis. The absorbance of the supernatant at 450 nm was measured using a microplate reader (MultiScan FC, Thermo Fisher Scientific K.K., Tokyo, Japan). The cell viability was calculated using Equation (5):

Cell viability (%) = [(A_s_ − A_b_)/(A_c_ − A_b_)] × 100 (5)

where A_s_ is the absorbance of the supernatant in the well with the sample, A_b_ is the absorbance of the supernatant in the well containing no cell with no sample, and A_c_ is the absorbance of the supernatant in the well containing cells with no sample.

### 2.13. Statistical Analysis

The results of flexural strength, Vickers hardness, degree of conversion, water sorption, water solubility, and cell viability were analyzed by means of the one-way analysis of variance (ANOVA) followed by Tukey’s post-hoc test using the EZR statistical software (Saitama Medical Center, Jichi Medical University, Tochigi, Japan). The one-way ANOVA was used, followed by the Tukey’s post-hoc test.

## 3. Results

### 3.1. Printability of the PMMA-Based Resins for the SLA 3D-Printing

**Figure 1:**
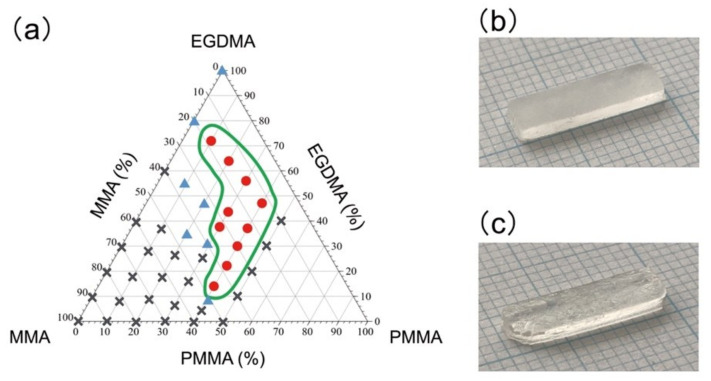
Printability of the PMMA-based resins for the SLA 3D-printing. (**a**) Phase diagram of the resin containing MMA, EGDMA, and PMMA. The symbols in the diagram denote printability; ○: More than 29 of the 36 printed samples were fabricated successfully, Δ: 4–28 per 36 samples were successful. ×: 3 or fewer of the 36 samples were successful. Photographs of (**b**) successful and (**c**) failed printed samples.

The printability of the PMMA-based resin was visually evaluated by the naked eye and by optical microscopy. The MMA, EGDMA, and PMMA components are represented in the trigonal phase diagram (Figure 1a). The printing was considered successful when all eight vertices in the rectangular sample were fabricated without any 3D-printing failures (Figure 1b). Meanwhile, we declared failure when a defect was found at any of the vertices (Figure 1c). A total of 36 rectangular samples were printed from each PMMA-based resin. When more than 29 out of 36 printed samples were successful, we concluded that the PMMA-based resins had a sufficient printability, indicated by ○ in the phase diagram (Figure 1a). PMMA-based resins from which 4–28 samples were successfully printed were deemed to have a lower printability, indicated by Δ in the phase diagram. In cases where three or fewer than 36 samples were successfully printed, we determined that the MMA-based resins lacked printability, indicated by × in the phase diagram (Figure 1a). The samples encompassed by the green line in the phase diagram were considered to be printable under the current printing conditions and were further characterized according to the following evaluations.

**Figure 2:**
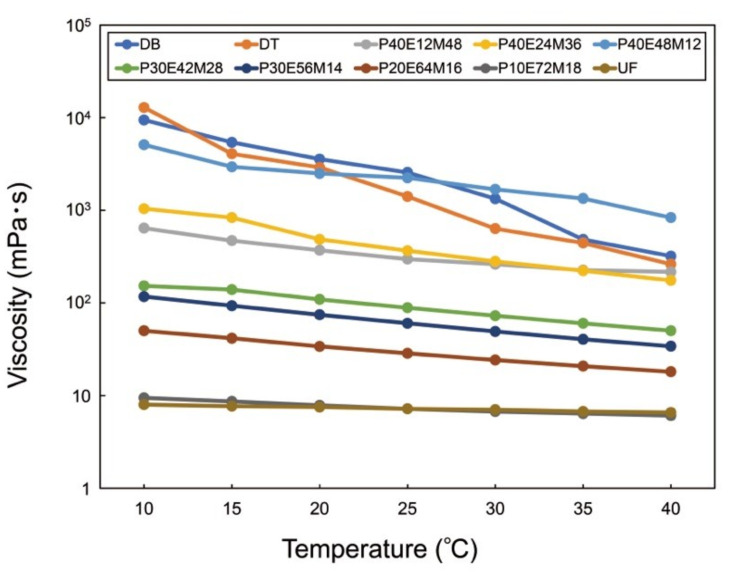
Viscosities of printable PMMA-based resins and commercial 3D-print resins (DB and DT). The printable PMMA-based resins did not contain photo-initiator to avoid unintentional curing by ambient light during the experiment.

The viscosity of the PMMA-based resin increased with increasing PMMA content and decreased with increasing ambient temperature (Figure 2). Higher concentrations of PMMA tend to form aggregates in the resin, significantly increasing their viscosity. The PMMA-based resins containing more than 50 wt% PMMA could not be printed owing to their high viscosity. With the exception of P40E48M12, the viscosities of the PMMA-based resins were lower than those of the commercial 3D-print resins (DB and DT) at the examined temperatures.

### 3.2. Mechanical Properties, Shear Bond Strength, and Degree of Conversion of Printable PMMA-Based Resins

**Figure 3:**
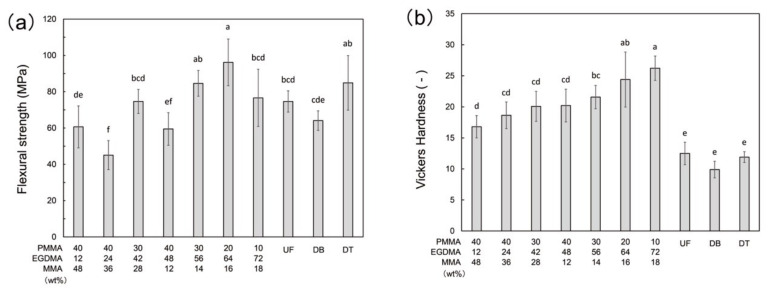
Mechanical properties of printable PMMA-based resins, conventional PMMA (UF), and commercial 3D-print resins (DB and DT); (**a**) flexural strengths, (**b**) Vickers hardness. The different letters indicate significant differences between the groups (*p* < 0.05, Tukey’s test, n = 10).

The flexural strength of PMMA-based resins increased with increasing EGDMA content (Figure 3a). Among the PMMA-based resins, the flexural strength P30E56M14 was significantly higher than that of DB and comparable to that of UF and DT. Furthermore, the flexural strength of P20E64M16 was significantly higher than that of UF and DF, and comparable to that of DT. The Vickers hardness of all PMMA-based resins were significantly higher than those of UF, DB, and DT, and increased with increasing EGDMA content (Figure 3b).

**Figure 4:**
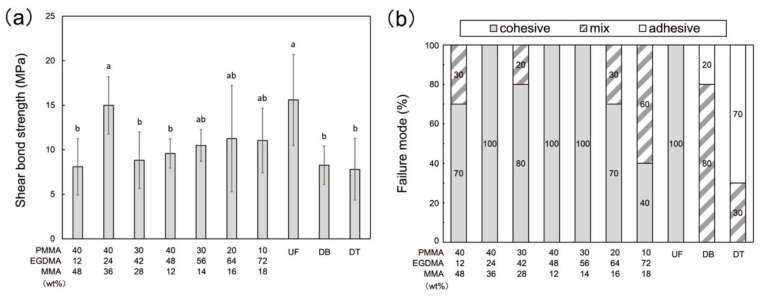
Bonding properties of printable PMMA-based resins, conventional PMMA (UF), and commercial 3D-print resins (DB and DT); (**a**) shear bond strength (SBS) of each sample and self-cured resin; and (**b**) sample failure modes. The different letters in the figure indicate significant differences between the groups (*p* < 0.05, Tukey’s test, n = 10).

The SBS of the P40E24M36, P30E56M14, P20E64M16, and P10E72M18 samples were comparable or significantly higher than those of the commercial resins (UF, DB, and DT) (Figure 4a). The failure mode (Figure 4b) indicates that the bonding strength of the sample increased in the order of adhesive, mixed, and cohesive failures. Only cohesive failure was observed in UF, suggesting that UF bonded strongly to the self-cured PMMA. Cohesive failure was the most common failure mode in each PMMA-based resin, while no adhesive failure was observed. In contrast, no cohesive failure was observed in the DB or DT samples. These results suggest that the PMMA-based resins have superior bonding properties to those of commercial 3D-print resins.

**Figure 5:**
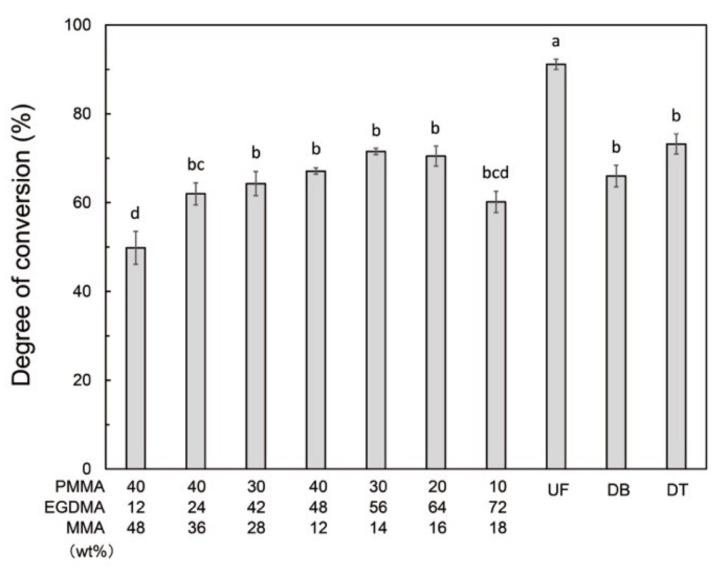
Degree of conversion of printable PMMA-based resins, conventional PMMA (UF), and commercial 3D-print resins (DB and DT). The different letters indicate significant differences between the groups (*p* < 0.05, Tukey’s test, n = 10).

The degree of conversion of the PMMA-based resins increases with increasing EGDMA content (Figure 5). With the exception of P40E12M48, the degree of conversion in the PMMA-based resins was significantly lower than that of UF, while comparable to that of DB and DT.

### 3.3. Physicochemical Properties and Cell Viability of the Printable PMMA-Based Resin

Based on the printability, mechanical properties, SBS, and degree of conversion, P30E56M14 was determined to be the optimal composition of the printed PMMA-based resin and subjected to further evaluation.

**Figure 6:**
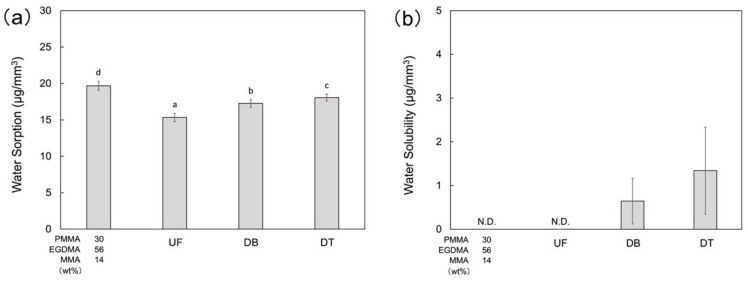
Physicochemical properties of the printable PMMA-based resin P30E56M14, conventional PMMA (UF), and commercial 3D-print resins (DB and DT); (**a**) water sorption; (**b**) water solubility. The different letters indicate significant differences between the groups (*p* < 0.05, Tukey’s test, n = 10).

The water sorption of the PMMA-based resin was significantly higher than the others (Figure 6a). However, the water solubility of the PMMA-based resin was lower than the detection limit (Figure 6b), indicating that monomer dissolution from the PMMA-based resin and UF was quite low.

**Figure 7:**
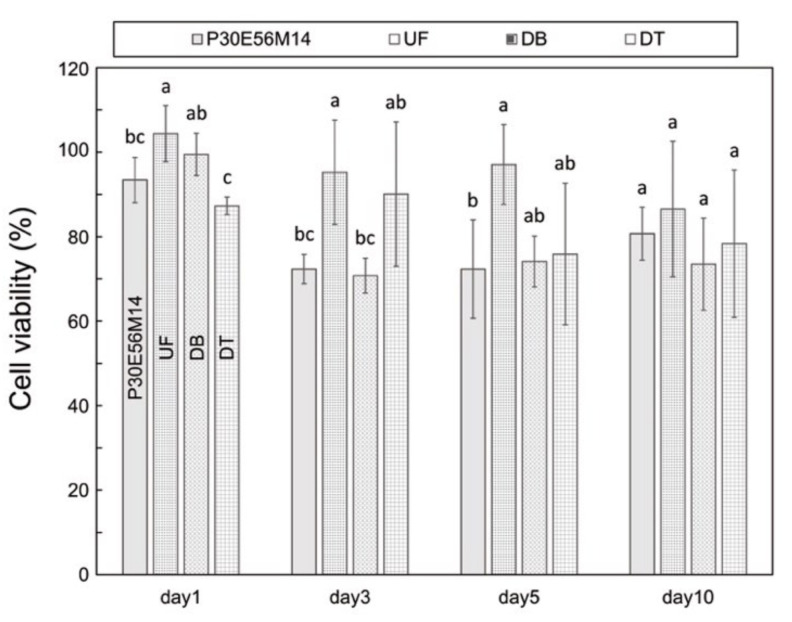
Cell viability in proliferation at specific days (1, 3, 5, and 10) of the printable PMMA-based resin P30E56M14, conventional PMMA (UF), and commercial 3D-print resins (DB and DT). The different letters indicate significant differences between the groups in each day (*p* < 0.05, Tukey’s test, n = 4).

The cell viability of the PMMA-based resin was significantly lower than that of UF on days 1, 3, and 5 (Figure 7). However, no significant difference among the examined samples was observed on day 10.

## 4. Discussion

Printable PMMA-based resins were prepared, and their properties were compared with three commercial resins: one conventional PMMA resin and two 3D-print resins. The properties of the printable PMMA-based resin were comparable to or greater than those of the commercial resins. The printable PMMA-based resins consisted of MMA, EGDMA, and PMMA, while the two commercial 3D-print resins, like commercial 3D-print resins on the market, contain multifunctional monomers (Table 2) [12,14]. The composition of the printable PMMA-based resins is more similar to that of conventional PMMA than those of commercial 3D-print resins.

The printability depended on the EGDMA and PMMA contents (Figure 1). The cross-linker is an essential component of the EGDMA composition. The cross-linker is typically an indispensable component of photocurable resin that ensures curing of the resins by light irradiation in the SLA 3D-printing process [30]. PMMA-based resins without EGDMA could not be printed under the current printing conditions. Because the monofunctional monomer MMA undergoes linear polymerization and does not form a three-dimensional network structure without any cross-linker, curing MMA in the SLA 3D-printing process is difficult. Pre-polymerized PMMA powder may help to cure the monomers in a similar way to the powder-liquid system adopted in conventional PMMA, where curing of the MMA monomer is facilitated by mixing with pre-polymerized PMMA powders. This powder-liquid system increases the degree of conversion and curing rate of the resin. Meanwhile, a higher PMMA content, especially above 40 wt%, significantly increased the viscosity of the PMMA-based resin. Such high-viscosity resins could not be printed at all by the SLA 3D-printer. A PMMA content of less than 50 wt% was therefore deemed most suitable.

The mechanical properties, flexural strength, and Vickers hardness, and degree of conversion increased with the EGDMA content of the resin (Figure 3). The cross-linker in EGDMA facilitated the polymerization of the PMMA-based resin. The strength of the polymer structure in the printed resin increased with the degree of conversion. These results are in agreement with the printability experiments, where the EGDMA was found to be an essential component to ensure resin curing by SLA 3D-printing. The PMMA component did not improve either the degree of conversion or the mechanical properties of the resin. However, EGDMA had a negative impact on the bonding properties (at least no improvement). A higher EGDMA content, particularly P10E72M18 and P20E64M16, caused adhesive failure in the SBS test, suggesting that the bonding strength declined owing to excessive EGDMA. Resins with an EGDMA content of 56% or less are considered to have sufficient bonding strength because cohesive failures were the main failure mode in the fractured samples. A linear polymer such as PMMA can form an interpenetrating polymer network (IPN) composed of two interlaced polymer networks. In dental materials, IPN structures can be found at the bonding interface between two PMMA-based resins, such as those found at the repaired fractures of a PMMA denture [35]. The IPN structure strengthens the bonding interface between the two resins owing to inter-diffusion and interlocking of the polymer chains. In the current study, both the printed PMMA-based resin and the self-cured resin consisted primarily of PMMA and thus formed an IPN structure at the bonding interface. Therefore, the bonding interface between the self-cured and printable PMMA-based resins was stronger than that between the commercial 3D-print resins. The bonding test indicate that the PMMA-based resin bonds strongly with other PMMA resins, such as the self-cured resin, which may allow the printable PMMA-based resin to be used as denture base (and teeth) and provisional crowns (and bridges). The strong bonding may be advantageous in denture repair because the fractured pieces can be easily bonded with the self-cured resin for repair. In clinical situations, repairing a denture is often preferred over replacing it because fabricating new dentures is expensive and requires several reservations [36]. Self-cured resins are typically used as repair materials owing to their low-cost and rapid repair properties. Commercial 3D-print resins exhibit poor bonding, resulting in the fracture of repaired dentures at the repair site [37,38]. Furthermore, the bond strength of the PMMA-based resin may facilitate shape reforming and adjustment using self-cured resins in provisional crowns and bridges. Commercial 3D-printed crowns can be used intraorally as provisional restorations owing to their mechanical properties [39,40], while repairing or relining the 3D printed provisional restoration remains a challenge. The current PMMA-based resins are potential candidates for overcoming this problem.

The water sorption of the PMMA-based resin was higher than that of conventional PMMA. This may be explained by the degree of conversion. The relatively low degree of conversion in the PMMA-based resin facilitates water sorption. The polymerization method is known to influence the degree of conversion and physicochemical properties: the physicochemical properties of heat-cured resins are superior to those of cold- (self-) cured resins [41,42]. Alternatively, the high water sorption of the PMMA-based resin may arise from the unique layer-by-layer structure of the 3D-printed object. When the 3D-printed object is immersed in water, the water can penetrate between the layers, giving the PMMA-based resin a much higher water sorption than that of conventional PMMA. However, the water sorption of the PMMA-based resin meets the criteria for the ISO standard 10477:2018 (dentistry—polymer-based crown and veneering materials), which stipulates that the water sorption value should be ≤40 μg/mm^3^ for clinical application. The water solubility of the printable PMMA-based resin was lower than the detection limit, strongly implying that the water solubility meets the ISO standard (≤7.5 μg/mm^3^).

The cell viability of the PMMA-based resin decreased over the first five days. This phenomenon may be related to the dissolution of components such as residual monomers of MMA and EGDMA; however, the amount of dissolution is quite low. Residual monomers dissolved in water and subsequently released into the surrounding environment reduce cell viability owing to cytotoxicity. The cell viability at day 10 exceeds 80%, which is comparable to conventional PMMA. Conventional PMMA are known to contain very few leached monomers and exhibit a low toxicity toward oral mucosal tissues [43,44,45,46]. The current findings imply that the cytotoxicity of the PMMA-based resin may be acceptable for use in an oral environment. The biocompatibility of the PMMA-based resins will be further clarified using in vitro cytotoxicity using human gingival cells [47] and in vitro allergy tests [48] in future work.

**Figure A1:**
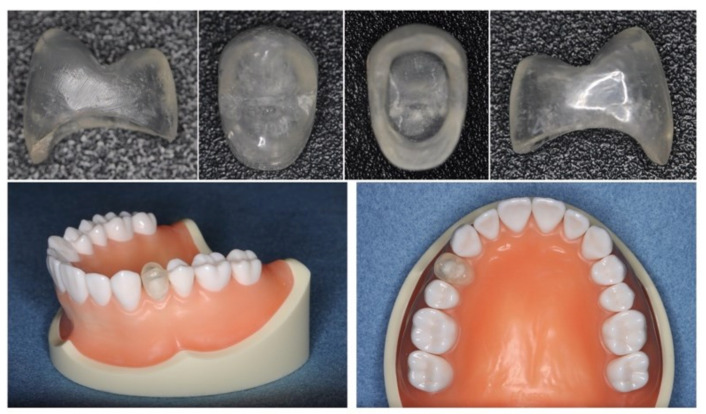
Photography of the 3D-printed crown set on the abutment tooth of the phantom model. The crown was fabricated using SLA 3D-printing and the PMMA-based resin P30E56M14. The printed crown surface was partially ground and polished to adjust the abutment tooth

The optimal resin can be printed to the temporary crown under the present 3D-printing conditions (Figure A1). However, this study did not investigate printing accuracy and trueness because these depend not only on the resin properties, but also on 3D-printer performance (light intensity, Z-Y-Z-stage mobility, etc.) and printing conditions (light-exposure time, layer thickness, printing direction, etc.) [49]. Such complex factors require individual investigation to fabricate an optimized object.

The present results indicate that the mechanical properties of the PMMA-based resin are comparable to or higher than those of the comparison samples of the commercial self-cured resin and 3D-print resins as well as the reported properties of commercial heat-cured PMMA resins [50,51]. This suggests that the present PMMA-based resin can potentially be employed in dental applications, such as temporary (and provisional) crowns and bridges, as well as denture-base and -teeth. Conversely, the mechanical properties of the PMMA-based resin are inferior to those of resin composites used for permanent crowns [52], implying that the PMMA-based resin is not suitable for long-term use in the oral cavity. Further improvement of the mechanical properties of PMMA resins through the addition of fillers will expand their applicability in dental prothesis.

## 5. Conclusions

The printable PMMA-based resin with composition of 30% PMMA, 56% EGDMA, and 14% MMA was developed for SLA 3D-printing. The printable PMMA-based resin had sufficient mechanical and physicochemical properties for clinical use and exhibited low cytotoxicity. The PMMA-based resin bound strongly to self-cured resin can be used for repair and bonding and is suitable for use in dental prostheses, such as temporary and provisional crowns and bridges, as well as denture-base and -teeth.

## Figures and Tables

**Table 1:** Reagents used for preparation of the PMMA-based resins.

Acronym	Reagent Name	Material Type	Product Company
**PMMA**	Polymethyl Methacrylate	Polymer	Sigma-Aldrich Co. LLC, Darmstadt, Germany
**MMA**	Methyl Methacrylate	Monomer	Fujifilm Wako Pure Chemical Corporation, Osaka, Japan
**EGDMA**	Ethylene Glycol Dimethacrylate	Cross-linker	Fujifilm Wako Pure Chemical Corp., Osaka, Japan
**BAPO**	Phenylbis(2,4,6-trimethylbenzoyl) phosphine oxide	Photo-initiator	Tokyo Chemical Industry Co., Ltd., Tokyo, Japan

**Table 2:** Commercial products used for control samples.

Acronym	Product Name	Material Type	Composition (%)	Product Company
**UF**	Unifast II Clear	Self-cured PMMA resin	Methyl methacrylate (90–100%), N,N-dimethyl-p-toluidine (1–2.5%), 2-(2H-benzotriazol-2-yl)-p-cresol (0.5–1%), Ethyleneglycol dimethacrylate (0.1–0.2%), hydroquinone (0.1–0.2%), poly(methyl methacrylate), others	GC, Tokyo, Japan
**DB**	Dima print denture base	3D-print resin for denture base	4,4′-isopropylidenediphenol, ethoxylated and 2-methylprop-2-enoic acid (40–60%), 7,7,9 (or 7,9,9)-trimethyl-4,13-dioxo-3,14-dioxa-5,12-diazahexadecane-1,16-diyl bismethacrylate (30–50%), Propylidynetrimethyl trimethacrylate (3–10%), Diphenyl(2,4,6-trimethylbenzoyl)phosphine oxide (<3%), Mequinol (<1%)	Kulzer Japan Co., Ltd., Tokyo, Japan
**DT**	Dima print denture teeth	3D-print resin for denture teeth		

**Table 3:** Compositions of the prepared PMMA-based resins. The sample name was abbreviated as follows: 30% PMMA-56% EGDMA-14% MMA as “P30E56M14”.

Composition (wt%)			
PMMA	MMA	EGDMA	BAPO
0	0	100	0.1
0	20	80	0.1
0	40	60	0.1
0	60	40	0.1
0	70	30	0.1
0	80	20	0.1
0	90	10	0.1
0	100	0	0.1
10	18	72	0.1
10	36	54	0.1
10	54	36	0.1
10	63	27	0.1
10	72	18	0.1
10	81	9	0.1
10	90	0	0.1
20	16	64	0.1
20	32	48	0.1
20	48	32	0.1
20	56	24	0.1
20	64	16	0.1
20	72	8	0.1
20	80	0	0.1
30	14	56	0.1
30	28	42	0.1
30	35	35	0.1
30	42	28	0.1
30	49	21	0.1
30	56	14	0.1
30	63	7	0.1
30	70	0	0.1
40	0	60	0.1
40	12	48	0.1
40	24	36	0.1
40	36	24	0.1
40	42	18	0.1
40	48	12	0.1
40	54	6	0.1
40	60	0	0.1
50	10	40	0.1
50	20	30	0.1
50	30	20	0.1
50	40	10	0.1
50	50	0	0.1

## Data Availability

The data presented in this study are available on request from the corresponding author.
